# Optical control of topological end states via soliton formation in a 1D lattice

**DOI:** 10.1515/nanoph-2024-0401

**Published:** 2024-10-22

**Authors:** Christina Jörg, Marius Jürgensen, Sebabrata Mukherjee, Mikael C. Rechtsman

**Affiliations:** Department of Physics, The Pennsylvania State University, University Park, PA 16802, USA; Physics Department and Research Center OPTIMAS, 26562 University of Kaiserslautern-Landau, Kaiserslautern D-67663, Germany; Department of Physics, Indian Institute of Science, Bangalore 560012, India; Department of Physics, Stanford University, Stanford, CA, USA

**Keywords:** discrete spatial solitons, topological photonics, pump-probe, Kerr-nonlinearity, waveguide lattices

## Abstract

Discrete spatial solitons are self-consistent solutions of the discrete nonlinear Schrödinger equation that maintain their shape during propagation. Here we show, using a pump-probe technique, that soliton formation can be used to optically induce and control a linear topological end state in the bulk of a Su–Schrieffer–Heeger lattice, using evanescently-coupled waveguide arrays. Specifically, we observe an abrupt nonlinearly-induced transition above a certain power threshold due to an inversion symmetry-breaking nonlinear bifurcation. Our results demonstrate all-optical active control of topological states.

## 1 Introduction

In topological systems, bulk invariants ensure robustness against local defects and disorder via protected states residing at the physical boundaries [1], [2], [3], implying the possibility of optical devices more robust to fabrication-induced disorder and damage [4], [5], [6]. While topological effects in linear systems have been well studied, nonlinear topological systems have only been explored very recently [7], [8], [9], [10], [11], [12], [13], [14], [15], [16], [17], [18], [19], [20], [21], [22], [23], [24], [25], [26], and a theoretical framework is just starting to emerge [27], [28]. Perhaps most understanding has been gained for nonlinear topological systems based on spatial solitons, which are self-consistent solutions of the nonlinear Schrödinger equation that maintain their spatial shape during propagation. At high optical power the nonlinear detuning of the refractive index (e.g. Kerr effect) balances the diffraction determined by the hopping between neighboring sites. Using waveguide arrays, spatial solitons [29], [30], [31], [32] have been observed in the bulk of anomalous Floquet topological insulators [14], [23], propagating along the edge [18], or being pumped by integer and fractionally quantized values in photonic Thouless pumps [19], [24], [33], [34].

Here, we use optical soliton formation to nonlinearly induce and probe a topological end state (that is, a 0D topological edge state) at any position in the bulk of a Su–Schrieffer–Heeger (SSH) lattice. In contrast to previous works that examined the role of nonlinearity on linear topological end states [35], [36], [37], [38], [39], [40], in our system the soliton optically induces a topological state in the bulk of the sample, not at a physical termination of the lattice. To this end, we use an all optical pump-probe setup with zero time delay and orthogonally polarized beams. We use the high-power pump beam to generate a soliton in the bulk of evanescently coupled waveguides that, in the limit of high power, effectively acts as a hard wall, while the low-power probe beam probes the end state that is induced next to the wall. The soliton therefore acts as an all-optical switch to turn on and off the topological (linear) end state. We theoretically show that the end state formation occurs above a certain threshold power due to a spontaneous inversion breaking nonlinear bifurcation. We complete our analysis by connecting the end states with previously (see Ref. [41]) known analytical solutions for dimers. While our experiments are carried out in optical waveguides, the concept is not limited to this platform, but can be transferred to other systems such as condensed matter, topological electric circuits, optomechanics, metamaterials, ultracold atoms etc.

## 2 Materials and methods

**Figure 1:**
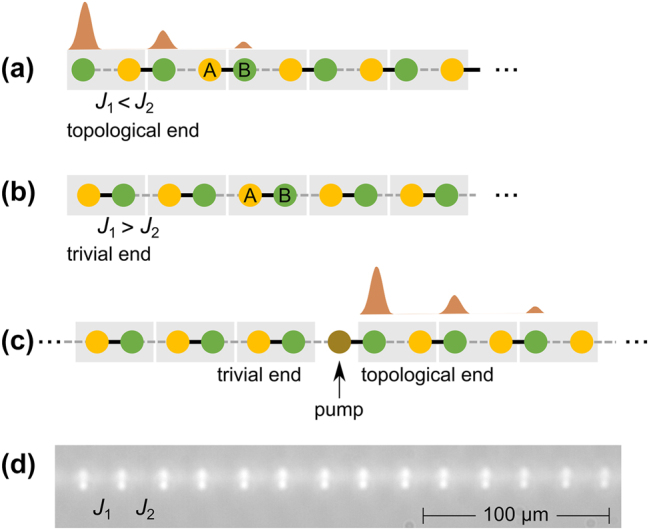
Schematic of the SSH lattice. (a) Topological termination for first hopping *J*_1_ < *J*_2_. (b) Trivial termination for first hopping *J*_1_ > *J*_2_. Boxes indicate the unit cells. (c) Nonlinear chain (in the infinite power limit) where the refractive index of the pumped site is being detuned, creating a topological ending to the right of the pumped site, while there is a trivial ending to the left of it. The sublattices are labeled A and B for easier identification, but do not have any (linear) onsite potential difference. (d) White-light image of the output facet of the SSH waveguide array.

We demonstrate our results using a SSH model – a dimer lattice, consisting of an A and B sublattice, with intra-cell hopping *J*_1_ and inter-cell hopping *J*_2_ (*J*_1_, *J*_2_ ≥ 0), depicted in Figure 1. For a finite chain, topological end states are present and exponentially localized provided the final hopping of the lattice is the weaker of *J*_1_ and *J*_2_. In coupled waveguide systems and at high optical power, nonlinearity emerges as an intensity-dependent modification of the waveguide’s refractive index (and therefore of the on-site potential, in contrast to nonlinear couplings [35], [42], [43]) due to the Kerr effect. The dynamics in our system for the pump and probe beam are then described by the following coupled equations:

(1) $$i\partial_{z}\Psi_{n}^{\text{pu}}=\sum_{m}H_{n,m}^{\text{lin}}\Psi_{m}^{\text{pu}}-g|\Psi_{n}^{\text{pu}}{|}^{2}\;\Psi_{n}^{\text{pu}}$$

(2) $$i\partial_{z}\Psi_{n}^{\text{pr}}=\sum_{m}H_{n,m}^{\text{lin}}\Psi_{m}^{\text{pr}}-g|\Psi_{n}^{\text{pu}}{|}^{2}\;\Psi_{n}^{\text{pr}},$$

where *H* ^lin^ is the tight-binding Hamiltonian of the linear SSH system, containing the hopping amplitudes *J*_1_ and *J*_2_, and $\Psi_{n}^{\text{pu}}\left(z\right)$ and $\Psi_{n}^{\text{pr}}\left(z\right)$ are proportional to the electric field amplitudes of the pump and probe beam at site *n* and propagation distance *z*. The total optical power $P_{\text{pu }\left(\text{pr}\right)}=\sum_{n}|\Psi_{n}^{\text{pu }\left(\text{pr}\right)}{|}^{2}$ in the pump (probe) polarisation is conserved. The nonlinear parameter *g* is defined as *g* = 2*πn*_2_/(*λA*_eff_), where *n*_2_ is the nonlinear refractive index, *A*_eff_ is the effective area of the waveguide mode, such that *gP*/*J*_1_ is dimensionless. Due to the vastly different powers of the beams with orthogonal polarizations we neglect the Kerr effect as induced by the probe beam, and only consider that of the pump. The pump’s dynamics (Eq. (1)) is described by the discrete nonlinear Schrödinger equation (corresponding to the Gross–Pitaevskii equation for the description of Bose–Einstein condensates and superfluids). The probe beam’s dynamics (Eq. (2)) is linear but dependent on the pump’s induced nonlinear potential. Therefore, we first focus on the formation of the soliton in the pump polarization at high optical power; later we will show its impact on the low-power orthogonally polarized probe beam.

**Figure 2:**
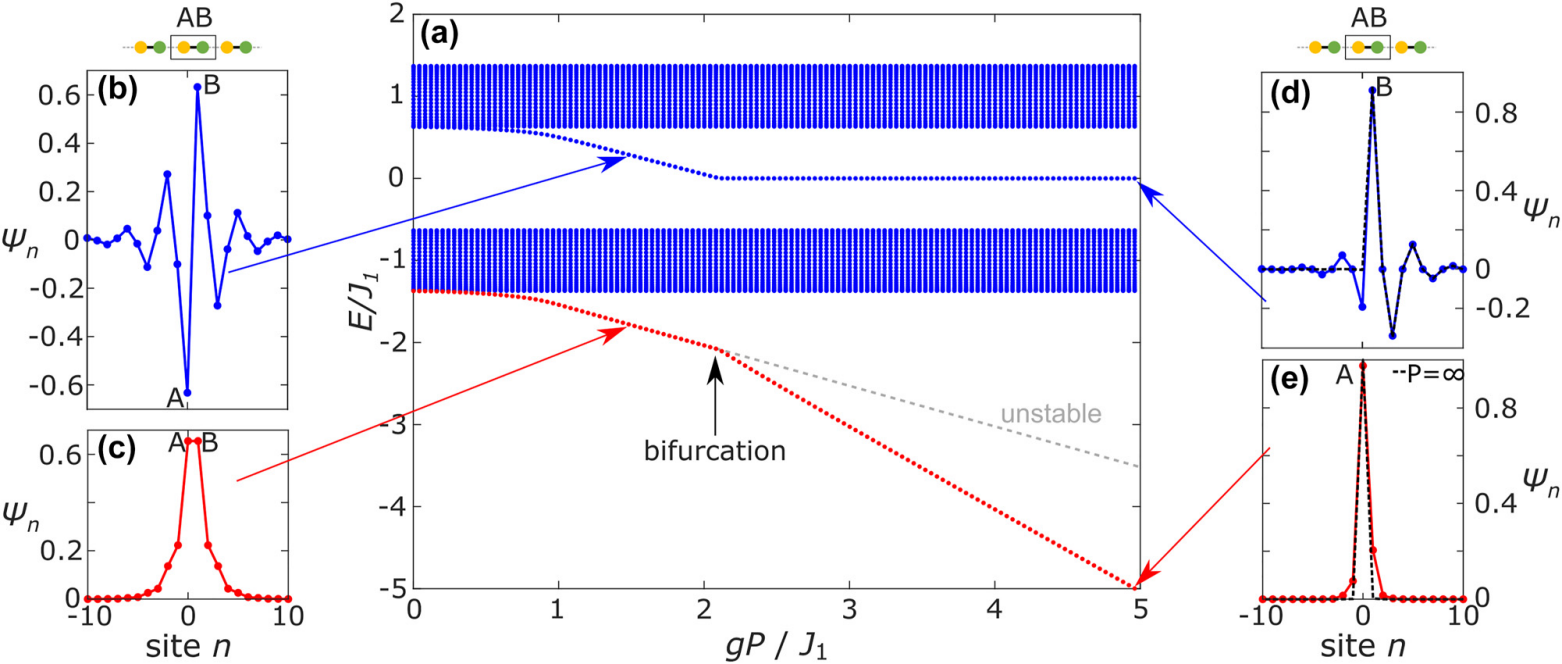
Spectrum obtained via the self-consistency method using 100 sites, *J*_2_/*J*_1_ = 0.37 and open boundary conditions with topologically trivial terminations. (a) The nonlinear eigenvalue of the soliton is plotted in red, and the eigenvalues of Eq. (2) under the potential of the obtained pump soliton wavefunction are plotted in blue. Clearly visible is the soliton bifurcation point at power *gP*/*J*_1_ ≈ 2, where the soliton eigenvalue changes its slope for increasing power, and the eigenvalue of the in-gap state goes to zero. This is the point where the symmetric soliton mode becomes unstable (gray dashed line; see also Supplementary Information Section 3 and Figure S2). The wavefunctions of the soliton (red, (c) and (e)) and the in-gap state (blue, (b) and (d)) are shown for low power (left) and high power (right). For low power, both states have equal support on the A and B sublattices. For *gP*/*J*_1_ ⪆ 2 the soliton symmetry is broken and the soliton has greater support on the input site (A in this case). The in-gap state then turns into a topological end state with support mostly on the B sublattice. The dashed black lines in (d) and (e) indicate the limit of infinite input power.

We solve Eq. (1) using the self-consistency method [44] to find localized soliton solutions. Figure 2(a) shows the calculated spectrum. The soliton energy eigenvalue is plotted in red, and blue dots show the eigenvalues of Eq. (2) under the potential defined by the pump soliton wavefunction. In other words, this calculation contains both the soliton itself, as well as the linear solutions that appear as a result of the presence of the soliton. We simultaneously observe two effects: For power *gP* ≈ 2*J*_1_ the soliton eigenvalue abruptly changes its slope as a function of power, and the in-gap state’s eigenvalue also abruptly approaches mid-gap (zero eigenvalue). The corresponding eigenstates (Figure 2(b)–(e)) reveal that for low power, the soliton wavefunction (red) has equal support on both sublattices, as the soliton wavefunction is inversion symmetric (see also Refs. [40], [45], [46], [47]), but at the transition, the soliton inversion symmetry is broken and gains more support on a single site for increasing power: The soliton undergoes a spontaneous inversion symmetry breaking nonlinear bifurcation (see Supplementary Information Section 3). After this bifurcation point the symmetric soliton becomes unstable (gray dashed line in Figure 2(a)) and therefore the corresponding in-gap state ceases to exist. Instead, a new stable soliton emerges with more support on one single site, and its corresponding in-gap state now has eigenvalues of zero for all *gP* ⪆ 2*J*_1_. Furthermore, the in-gap state’s support on the A sublattice becomes very small by comparison above the threshold. This is highly reminiscent of a topological end state in the SSH lattice: In systems that respect chiral symmetry, localized zero modes are supported on only one sublattice. Note that, in the limit of infinite power the soliton wavefunction only has support on a single site and the wavefunction of the linear in-gap state exactly matches that of a topological end state (dashed black lines in Figure 2(d) and (e)). We understand this behavior as follows: In this limit, the pump light detunes the input waveguide’s refractive index sufficiently strongly that it effectively acts as a wall, cutting the SSH lattice into two subchains (see Figure 1(c)), with a trivial termination on the left and a topologically non-trivial termination on the right. Note that the effect observed here is distinctly different from the case of linearly detuning the refractive index of a single site in the bulk, though: In the case of linear detuning, the in-gap state only gradually and asymptotically approaches zero eigenvalue, while for the end state induced by soliton-formation on the neighboring site the transition of its eigenvalue to zero is rather sharp when the power crosses the bifurcation threshold (see also Supplementary Information).

The fully dimerized limit of the lattice (i.e., when *J*_2_ = 0) allows us to understand this sharp transition around power *gP* ≈ 2*J*_1_. In this regime, we may consider the “lattice” as being simply composed of individual dimers (i.e., pairs coupled sites that are uncoupled to others). For a single dimer, for *gP* < 2*J*_1_, one symmetric and one anti-symmetric nonlinear mode exists. However, for *gP* > 2*J*_1_ the symmetric mode becomes unstable through a symmetry-breaking nonlinear bifurcation (see also Supplementary Information Section 3 for a linear stability analysis) and a new stable nonlinear mode emerges that has dominant support on one of the two dimer sites [41]. The linear eigenstate of Eq. (2) that includes the potential induced by the soliton then has an eigenvalue of exactly zero (see Supplementary Information Section 3). When moving away from the dimerized limit, these become the localized soliton mode and the mid-gap end state, respectively. As we show in Supplementary Information Figure S7, as *J*_2_ is increased from zero, the transition remains, but shifts to slightly higher power. In our experiment, we use a pump beam to induce the soliton, which then defines the trapping potential for the linear end state, which we probe in the orthogonal polarization. Note that in the experiment only stable modes are accessible.

## 3 Results: experiment

We experimentally probe the soliton-induced end state in the bulk of a nonlinear SSH chain using single-moded, femtosecond laser written waveguides in Corning Eagle XG borosilicate glass (for more details on the fabrication method see the Supplementary Information Section 1 and Ref. [14], [48]). To maximize the effective nonlinearity (*gP*/*J*_1_), we separate the waveguides such that the evanescent couplings *J*_1_ and *J*_2_ are small, while also keeping the coupling constants large enough to see enough transverse dynamics over the maximum propagation length of 150 mm. The experimental parameters are: *J*_1,pr_ ≈ (0.039 ± 0.006) mm^−1^ and *J*_2,pr_ ≈ (0.016 ± 0.003) mm^−1^ for the probe beam, and *J*_1,pu_ ≈ (0.022 ± 0.004) mm^−1^ and *J*_2,pu_ ≈ (0.008 ± 0.002) mm^−1^ for the pump beam; the hoppings for the pump and probe beam are different because the polarizations are different. *gP*/*J*_1_ is then scaled to *J*_1,pu_ (dropping the index pu), since the pump power determines the bifurcation threshold. In order to reach the necessary degree of nonlinearity, we use high power laser pulses (see Supplementary Information Section 2).

The experimental setup is depicted in Supplementary Figure S1. We use a pump-probe scheme, in which we split the emitted pulses from a single laser source into two orthogonally polarized beams, one with high and the other with low power. We use the high-power beam as the pump beam that excites a soliton, and simultaneously probe the induced end state using a weak-power probe beam in the neighboring waveguide. Both beams are focused into single waveguides using the same lens. Temporal overlap of the pump and probe beam is assured by adjusting the probe beam path with the help of a delay stage. Finally, we image the waveguide intensities at the output facet of the orthogonally polarized pump and probe beam onto separate cameras, using a thin film polarizer. In the experiment, the power of the probe beam is fixed at approximately 0.07 times the pump beam power and therefore is always low enough to ensure that it does not generate any nonlinear effects itself. We estimate the nonlinear length – the propagation distance for which nonlinear effects are significant – for the probe beam at its highest power to be 1/*g* = 173 mm, which is larger than the length of our sample.

**Figure 3:**
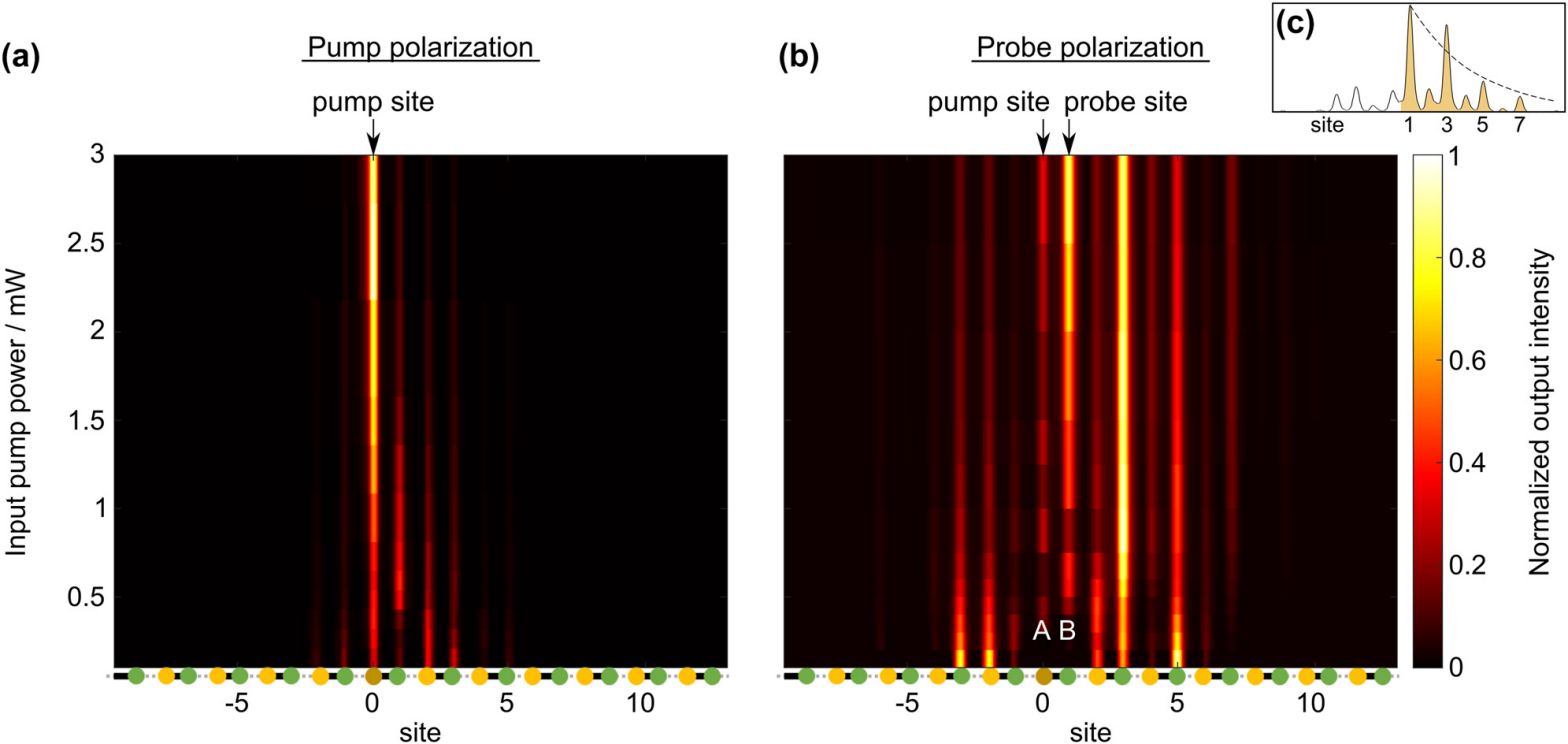
Experimental observation of nonlinearly-induced topological end states. (a) Pump intensity at the output facet for increasing pump power. The pump beam is injected into site 0. (b) Probe intensity at the output facet for increasing pump power. The pump beam is injected into site 0, while the probe beam is focused into the neighboring strongly-coupled site (site 1). For low power the probe intensity shows equal support on both sublattices, while for high power we see a stronger support on the B sublattice. The inset (c) shows the intensity profile (solid line) for the highest power reached in experiment, along with a fit for exponential decay of the intensity towards the bulk (dashed line).

We focus the pump beam into a waveguide in the lattice (indexed by site 0 in Figure 3(a)) and the probe beam into the strongly coupled neighboring site, waveguide 1. Figure 3(a) shows the output intensity distribution for the pump beam as a function of increasing input power (see also Eq. (1)). For low input power the intensity is distributed over multiple sites. At high power the wavefunction is strongly peaked on a single site as the symmetry-broken soliton forms and is efficiently excited due to the large mode overlap with a single-site excitation. The soliton acts as the hard wall to split the SSH chain. Note that due to the single-site excitation, we do not efficiently excite the symmetric soliton at low power.

Figure 3(b) shows the output intensity distribution of a co-propagating (zero time-delay) low-power probe beam under the influence of the pump beam as a function of the pump input power (see also Eq. (2)). For low pump power we observe probe beam intensities on the left and right of the pump waveguide, as expected for the excitation of extended, dispersed bulk states. With increasing pump input power, the power on the left of the pump beam decreases towards zero, as well as the power on the A sites to the right of the pump beam. This transition coincides with the pump forming the highly localized soliton, detuning the input site to form the ‘wall’ between the left and right sides. At the highest pump input power that we can reach in our experiment (without damaging the sample), for which the wall is the most pronounced, we observe that the probe beam is populating B sites with more intensity than A sites, a hallmark of an SSH end state. Repeated measurements for different input waveguides show a similar behavior (see Supplementary Information Figure S10). Note that the power threshold for the soliton bifurcation depends very sensitively on in-coupling efficiency, the slight refractive index differences among the waveguides, as well as their losses. Therefore, the induced SSH end state appears at different power thresholds for each measurement (see Supplementary Information Section 8 for more details).

## 4 Discussion

**Figure 4:**
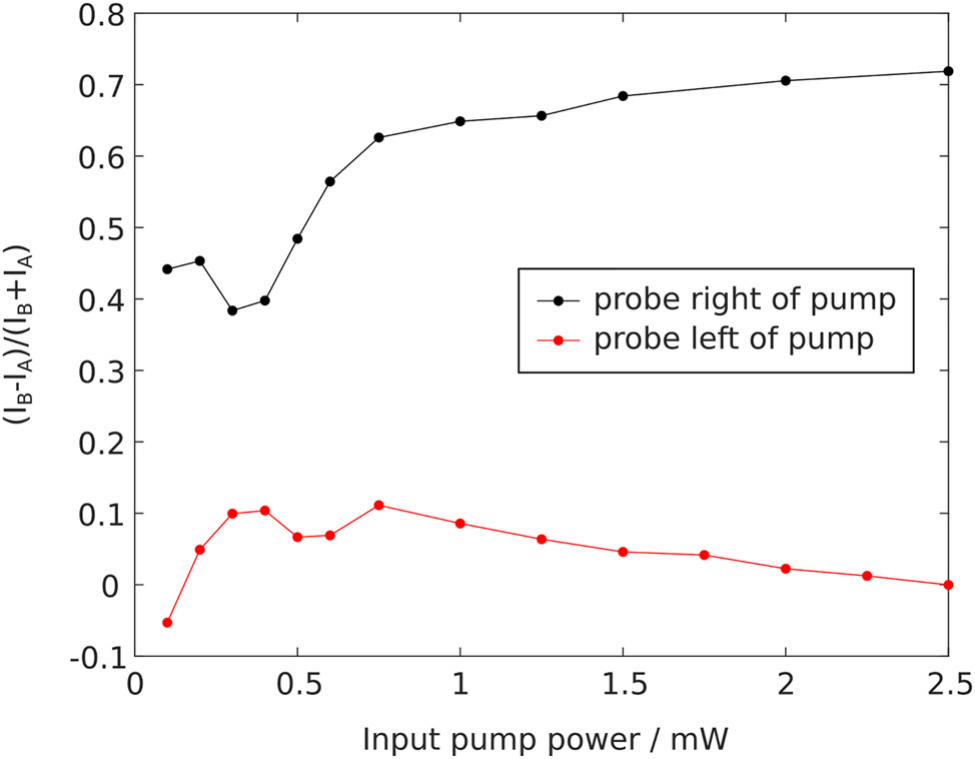
Intensity imbalance in the formation of topological end states. Extracted intensity imbalance between A and B sublattice (*I*_B_ − *I*_A_)/(*I*_B_ + *I*_A_). Black dots: Probe beam is injected to the right of the pump (waveguide 1), i.e., the configuration shown in Figure 3(a), exciting an induced topological end state. Red dots indicate the imbalance when the probe is injected to the left of the pump (waveguide −1), i.e., not exciting a topological end state. Lines are guides to the eyes.

To quantitatively evaluate the end state formation, we analyze the intensity imbalance (*I*_B_ − *I*_A_)/(*I*_B_ + *I*_A_) of the probe beam for the intensity *I*_A_ (*I*_B_) in the A (B) sites to the right of the probe beam for the measurements shown in Figure 3(b), as shown in Figure 4. In the SSH model, chiral symmetry dictates that all bulk eigenstates have equal support on both sublattices, i.e., the imbalance for all bulk states is zero. By contrast, the topological end states at mid-gap have support on only one of the two sublattices. While a linear superposition of bulk states may be imbalanced, this will vanish with increasing propagation distance. A large imbalance therefore indicates the excitation of a topological end state. Note that although the soliton locally breaks chiral symmetry, chiral symmetry is still approximately present in the bulk – see Supplementary Information Section 5. Figure 4 shows a strong increase in the imbalance for increasing input pump power, signaling the creation of the topological end state due to the nonlinearly-induced hard wall. In contrast, we also probe the waveguide to the left of the pumped waveguide, site −1, i.e., its weakly coupled neighbor, which cannot host a topological end state (see Figure 1(c)). The output intensity is plotted in Supplementary Figure S9 and the extracted imbalance (red line in Figure 4) remains around zero even for increasing pump power. This serves as an experimental control and confirms that the soliton induces an end state on its neighboring strongly-coupled site.

## 5 Conclusions

In conclusion, we have shown theoretically and experimentally that the inversion symmetry-breaking nonlinear bifurcation that forms a soliton peaked on a single site in a nonlinear SSH lattice, spontaneously induces a topological end state on its neighboring strongly-coupled site. This work demonstrates the active formation and control topological states all-optically, anywhere in the bulk of the system.

## Supplementary Material

Supplementary Material Details
