# Supplementary material for: Optical control of topological end states via soliton formation in a 1D lattice
Source: Nanophotonics. 2024 Oct 22;14(6):769–75. doi: 10.1515/nanoph-2024-0401 (PMC11964185; doi:10.1515/nanoph-2024-0401)
Supplement: Supplementary file 1 — Supplementary Material Details [file j_nanoph-2024-0401_suppl_001.pdf]

Supplementary Information: Optical control of topological end states via soliton formation in a 1D lattice

Christina Jörg,[1,2] Marius Jürgensen,[1] Sebabrata Mukherjee,[1,3] and Mikael C. Rechtsman[1]

[1]*Department of Physics, The Pennsylvania State University, University Park, Pennsylvania 16802, USA*
[2]*Physics Department and Research Center OPTIMAS, University of Kaiserslautern-Landau, Kaiserslautern D-67663, Germany*
[3]*Department of Physics, Indian Institute of Science, Bangalore 560012, India*

(Dated: October 17, 2024)

I. FABRICATION

The waveguide arrays were fabricated in Corning Eagle XG borosilicate glass by femtosecond laser direct writing. We used a Menlo BlueCut fiber laser with a wavelength of 1030 nm and repetition rate of 500 kHz. The average laser power for the fabrication of the waveguides was around 225 mW in the laser focus and the sample on the stage was translated at 8 mm/s through the laser focus. We shaped the beam by the use of a slit with width of 1.8 mm to control the elliptical cross-section of the waveguides (for more details see Supplementary Information section of reference [1]).

II. MEASUREMENT SETUP

The measurement setup is depicted in Fig. S1 and shows a pump-probe setup in which pump and probe polarization can be tuned individually. A pulsed laser beam (Menlo BlueCut) is sent through a Glan Thompson polarizer which allows the power to be tuned, and is polarized horizontally by a wire-grid polarizer (not shown in Fig. S1). The beam has a wavelength of 1030 nm and pulse duration of approximately 450 fs at a repetition rate of 10 kHz. The beam is then split by a 90:10 non-polarizing beamsplitter cube into two paths, the probe path (light red), and the pump path (dark red). The polarization of the beam in the probe path is rotated by 90° by a half-waveplate. The beam in the probe path is retro-reflected by a prism mounted onto a delay stage, which allows to overlap the pulses of the pump and probe beam temporally by adjusting the probe beam's path length. A second 50:50 non-polarizing beamsplitter cube combines the pump and probe beam again, before an aspheric lens ($f = 18.40$ mm, Thorlabs C280TMD-B) focuses the two beams into neighboring waveguides. The output intensity of the waveguides' output facet is imaged by a lens ($f = 25.4$ mm, Thorlabs LB1761-B-ML), and we use a thin film polarizer under the Brewster angle to separate the pump and probe beam polarizations, such that they are imaged onto two separate CMOS-cameras (Thorlabs DCC1545M). Since the polarizer is not perfect, a small amount of pump beam polarization remains leaking through onto the probe camera, which is non-negligible given that the pump beam intensity is significantly greater than the probe. After blocking the residual pump polarization using an additional linear polarizer, the resulting pump polarization leaking through to the camera increases with pump power, but is smaller than 10% up to an input power of 2 mW, and reaches approximately 20% for an input power of 3 mW. The increase in leakage with pump power points to polarization changes due to a nonlinear process.

We observe spectral broadening of the pump pulses due to self-phase modulation in the waveguides to about 70 nm FWHM at 2 mW input power after 150 mm of propagation in the glass waveguides.

Due to the elliptical cross-section of the fabricated waveguides, the coupling constants between neighboring waveguides is a function of polarization, and thus different for pump and probe beam. The values for the coupling constants for the different polarizations are extracted from measurements on simple integer lattices and used in the simulations.

III. LINEAR STABILITY ANALYSIS

We conduct linear stability analysis, following reference [2], to check the stability of the soliton solutions found by the self-consistency method. The solution Ψ of the nonlinear Schrödinger equation with eigenvalue E_0

$$E_0 \Psi = H_{\mathrm{lin}} \Psi - g|\Psi|^2 \Psi \tag{S1}$$

is of the form $\Psi(t) = \Psi(x) \exp(-\mathrm{i} E_0 t)$. Note that $\Psi = \begin{pmatrix} \Psi_1 \\ \Psi_2 \\ \vdots \\ \Psi_N \end{pmatrix}$ is a vector containing the electric field amplitudes at the waveguide sites, and $g|\Psi|^2 = \begin{pmatrix} g|\Psi_1|^2 & & & \\ & g|\Psi_2|^2 & & \\ & & \ddots & \\ & & & g|\Psi_N|^2 \end{pmatrix}$ represents a diagonal matrix. We add a small perturbation to this solution

$$\Psi(t) = (\Psi(x) + \epsilon(v + \mathrm{i}w)) \exp(-iE_0 t). \tag{S2}$$

Inserting Eq. S2 into Eq. S1, neglecting higher order terms in ϵ ($\mathcal{O}(\epsilon^2)$) and sorting for real and imaginary

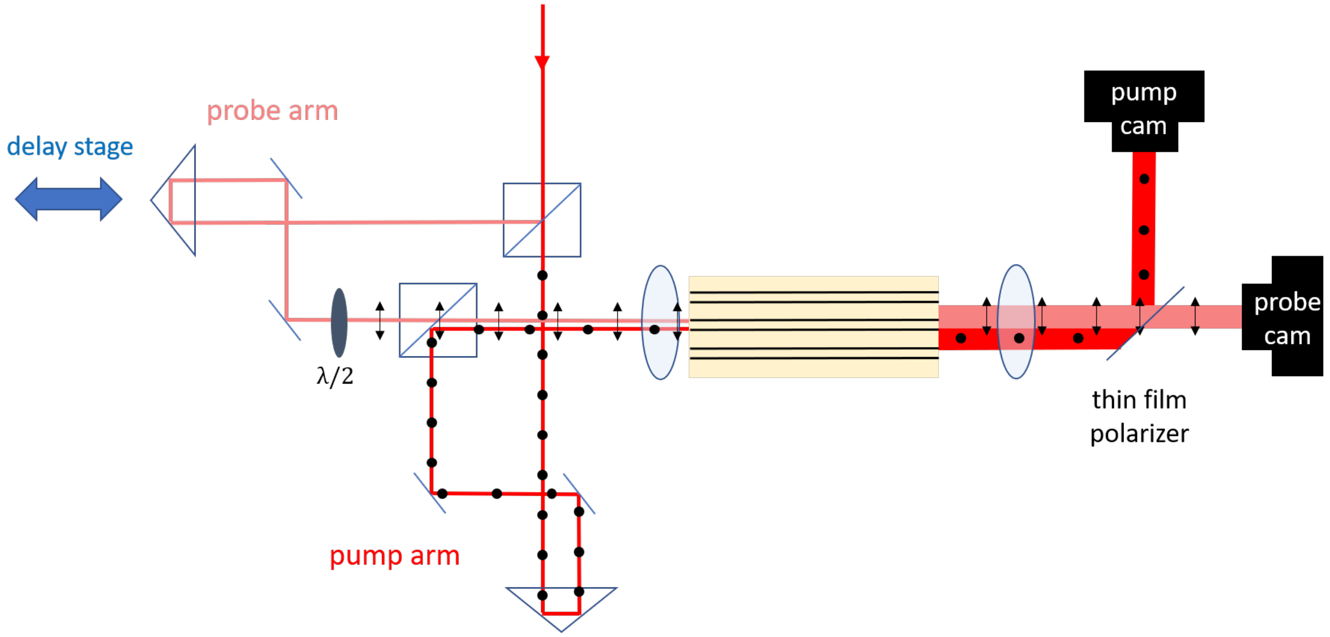

FIG. S1. **Measurement setup.** A strong pump beam excites the soliton in the waveguide array, while a weak probe beam with orthogonal polarization probes the induced topological end state.

parts yields

$$\partial_t w = -(-E_0 - 2g|\Psi|^2 - g\Psi^2 + H_{\text{lin}})v = -L_+ v \qquad \text{(S3)}$$

and

$$\partial_t v = (-E_0 - 2g|\Psi|^2 + g\Psi^2 + H_{\text{lin}})w = L_- w. \qquad \text{(S4)}$$

We separate time and space variables

$$v(x,t) = \tilde{v}(x)\exp(\lambda t) \qquad \text{(S5)}$$
$$w(x,t) = \tilde{w}(x)\exp(\lambda t) \qquad \text{(S6)}$$

and thus Eq. S3 and S4 result in an eigenvalue problem

$$\lambda^2 \tilde{v} = -L_- L_+ \tilde{v}. \qquad \text{(S7)}$$

We see that eigenvalues of $-L_- L_+$ with $\lambda^2 > 0$ imply unstable soliton solutions Ψ.

Figures S2(a),(b) show the eigenvalue spectra colored in terms of the linear stability eigenvalues of Eq. S7, where gray dots mean that the soliton is unstable ($\lambda^2 > 0$). Shown are the case of a single dimer (a), i.e. $J_2 = 0$, and the SSH lattice with $J_2/J_1 = 0.37$ (b). For the single dimer in (a), we see that for low power a stable symmetric nonlinear eigenstate exists with equal phase and amplitude on both dimer sites (denoted by $\upuparrows$ and red dots):

$$\Psi^{\text{pu}}(x) = \sqrt{P/2}\begin{pmatrix} 1 \\ 1 \end{pmatrix} \qquad \text{(S8)}$$

The eigenvalues of the probe states (the solutions of Eq. 2 in the main text) are plotted in blue, and correspond to the state

$$\Psi^{\text{pr}}(x) = \sqrt{P/2}\begin{pmatrix} 1 \\ -1 \end{pmatrix}. \qquad \text{(S9)}$$

At the bifurcation point, the symmetric soliton becomes unstable for $gP/J_1 > 2$ (lower gray dots) and therefore the corresponding probe state ceases to exist (upper grey dots). Instead, a new stable nonlinear eigenstate emerges with more support on one site than the other (denoted by $\uparrow\bullet$ and orange stars)

$$\Psi^{\text{pu}}(x) = \sqrt{P/2}\begin{pmatrix} \sqrt{1+\sqrt{1-(2J/gP)^2}} \\ \sqrt{1-\sqrt{1-(2J/gP)^2}} \end{pmatrix} \qquad \text{(S10)}$$

[3], and its corresponding probe state (light blue stars)

$$\Psi^{\text{pr}}(x) = \sqrt{P/2}\begin{pmatrix} -\sqrt{1-\sqrt{1-(2J/gP)^2}} \\ \sqrt{1+\sqrt{1-(2J/gP)^2}} \end{pmatrix} \qquad \text{(S11)}$$

has eigenvalue $E = 0$ for all $gP/J_1 > 2$, as one can easily check when inserting the states in Eq. 2 in the main text. A very similar bifurcation exists for the SSH lattice with $J_2/J_1 = 0.37$ in Fig. S2(b): The symmetric soliton becomes unstable near $gP/J_1 = 2$, and the new stable soliton has more support on a single site $\uparrow\bullet$ (see also Refs. [4, 5]). The corresponding in-gap state then also changes to have $E = 0$. This provides a direct link between the extreme dimer case in Fig. S2(a) and the SSH case in Fig. S2(b) - both occur due to an inversion-symmetry breaking bifurcation. In the SSH case, this allows for the formation of an effective topological end next to the site with the soliton peak (see Fig. S3).

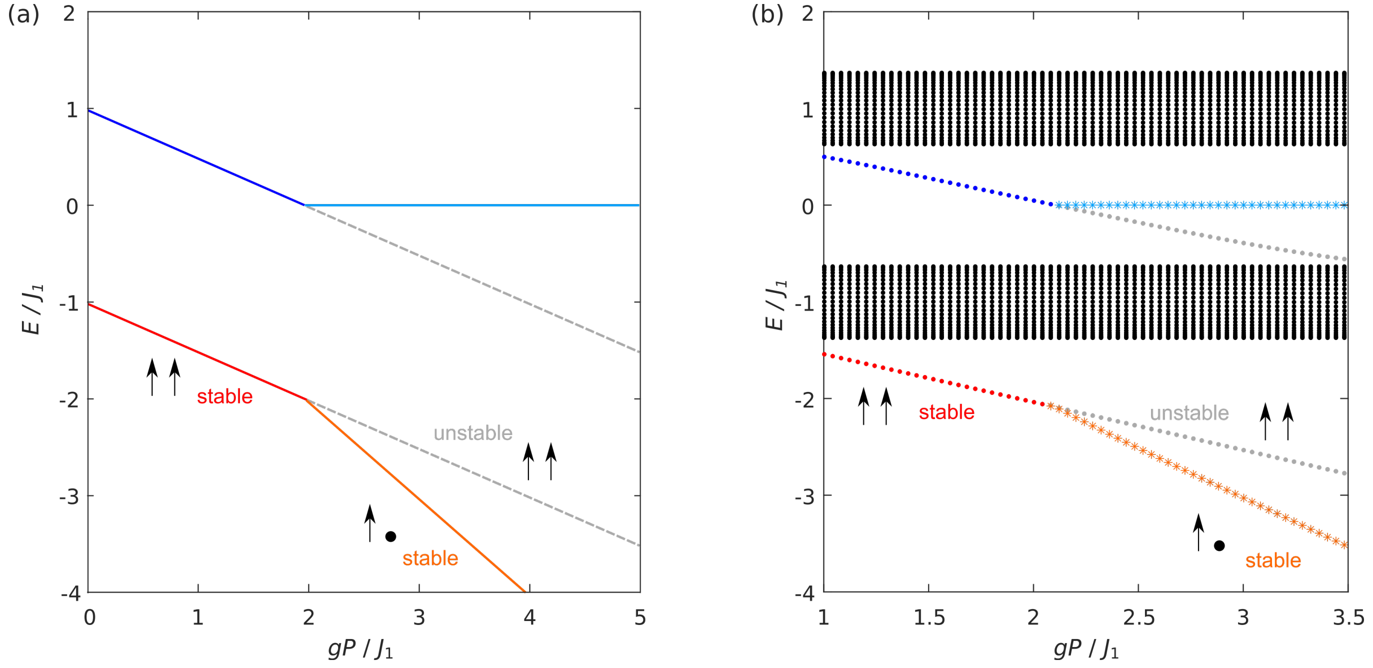

FIG. S2. **Linear stability analysis.** Eigenvalue diagrams as a function of power for the case of a single dimer (a) and the SSH lattice with $J_2/J_1 = 0.37$ (b). Unstable soliton eigenvalues (with their corresponding in-gap states) are shown in gray. The symmetric soliton (denoted by $\uparrow\uparrow$ and red dots) is only stable up to power $gP/J_1 < 2$ (the bifurcation point). For larger power, this symmetric soliton becomes unstable and therefore the corresponding in-gap state ceases to exist (gray dots). Instead, a new stable soliton emerges with more support on one single site (denoted by $\uparrow\bullet$ and orange stars), and its corresponding in-gap state (light blue stars) now is at $E = 0$ for all $gP/J_1 > 2$.

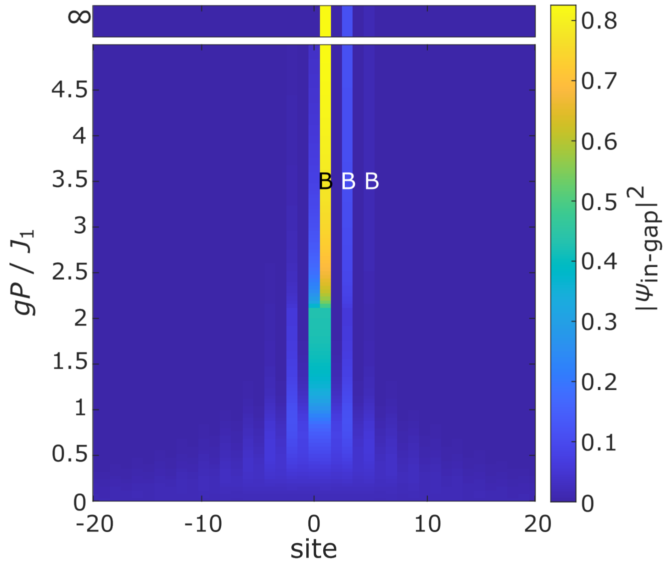

FIG. S3. **Numerical simulations of the intensity of the in-gap state** $\psi_{\text{in-gap}}$ as a function of power, as obtained via the self-consistency method. The letters in the plot indicate sublattice B.

IV. FURTHER SOLITONS IN THE SSH LATTICE

In Fig. S4 we show the stable soliton states in the SSH lattice along with their induced linear states. In general, below the bifurcation point, i.e. for $gP/J_1 \lessapprox 2$, there exist two stable solitons modes: an anti-symmetric one $\uparrow\downarrow$ (shown in magenta, with eigenvalues in the finite gap) and a symmetric one $\uparrow\uparrow$ (shown in red, with eigenvalues in the semi-infinite gap below the lowest bulk bands). Above the bifurcation point, i.e. for $gP/J_1 \gtrapprox 2$, the symmetric soliton becomes unstable and an asymmetric soliton $\uparrow\bullet$ emerges. This means that above the bifurcation point, the stable solutions are the asymmetric soliton $\uparrow\bullet$ (orange), as well as the anti-symmetric soliton $\uparrow\downarrow$ (magenta; that becomes unstable when its eigenvalues coincide with the bulk bands, shaded grey area). Both induce "defects" for the probe polarization: In green the symmetric defect induced by the anti-symmetric soliton is shown and in cyan the topological end state induced by the asymmetric soliton is shown. While these states are not orthogonal, note that the amplitude of the green defect outside the excited dimer is quite small, even for $gP/J_1 = 2.5$. This means that when we pump one lattice site, there is some small overlap with the anti-symmetric soliton and we might excite it to some proportion. However, since the green defect has negligible amplitude outside the excited dimer, the induced topological end state

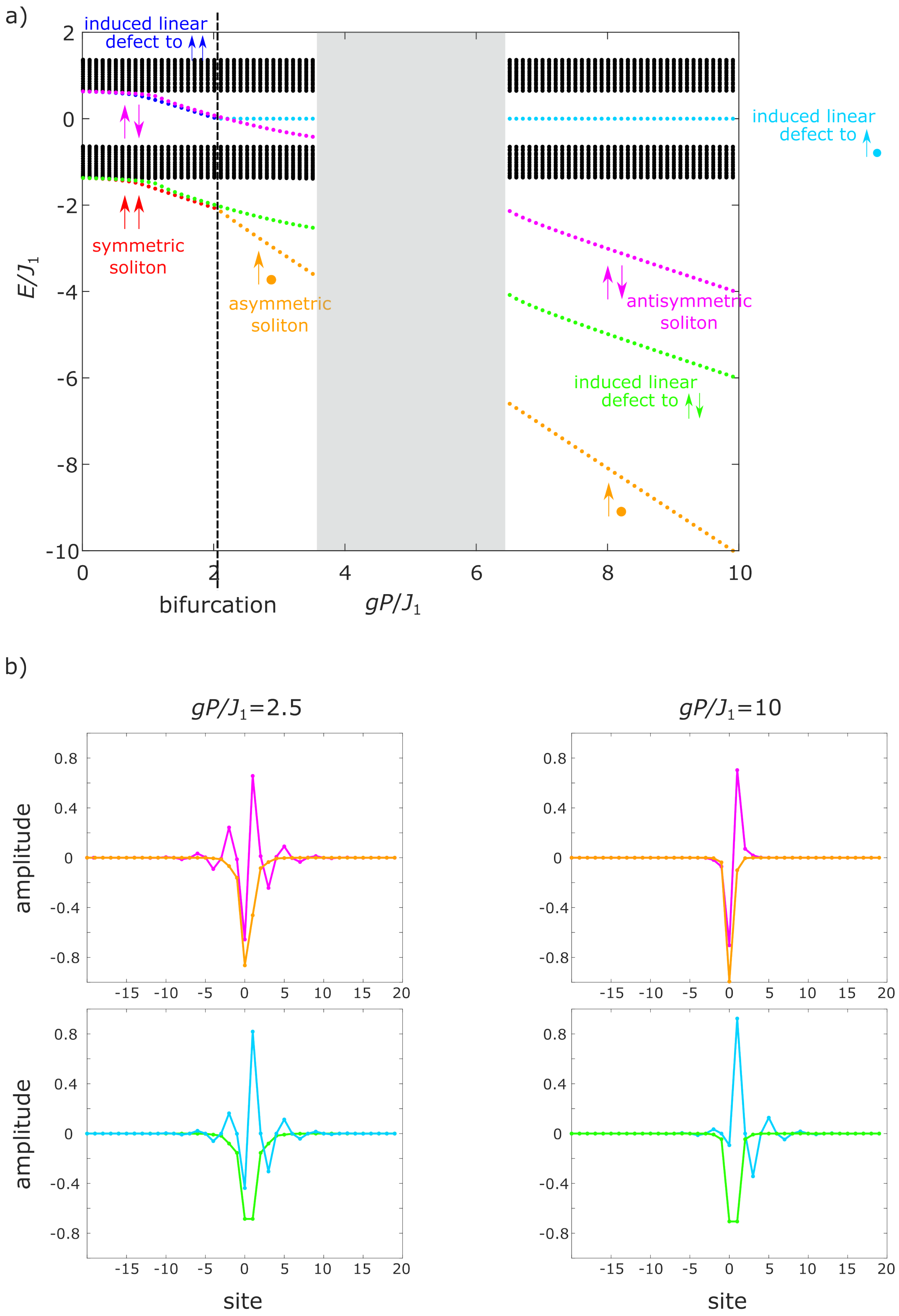

FIG. S4. **Stable solitons and their induced linear defects.** Mode profiles above the bifurcation point are shown in the lower panels.

(cyan) can still be faithfully sampled by the probe beam.

We also note that the asymmetric soliton is not exclusively localized only on one site. This is only true in the limit of infinite power. The remaining power on the second dimer site can be viewed as a weak defect at the end of the "cut" chain (compare Fig. 1(c) in the main text) which leads to a perturbation of the topological end state. In our case, the deviation of the linear eigenvalue from zero (compared the band gap) is a good indication of the perturbation of the end state. As can be seen in Fig. S2, this effect is negligible. Hence, the contrast between the sublattices is extremely well preserved. Furthermore, as can be seen for the cyan defect mode (the induced topological end state) for $gP/J_1 = 2.5$ in Fig. S4, as well as in the intensity profile for long propagation distances in Fig. S5, the effect on the form of the induced topological state is negligible. For $gP/J_1 = 2.5$ (cyan mode plotted in the left bottom panel of Fig. S4) the amplitudes on site -1 and site 2 (i.e. the A-sites) are below 0.002. Thus, it seems that the defect at the end of the "cut" chain is sufficiently weak to not influence the induced topological end state very much.

We also show simulation results of Runge-Kutta simulations of Eq. (1) and (2) of the main text for longer propagation distances and a power above the bifurcation threshold. The pump beam is focused into site 0 (upper panel) and the probe beam samples site 1 (lower panel). Even for long propagation distances the end state survives and is clearly visible, since much of the stray light (due to non-perfect excitation of the end state with the single-site probe beam) has moved outwards. Unfortunately, in the experiments we are limited in sample length; however, the induced topological end state is still clearly visible in our measurements.

V. APPROXIMATE CHIRAL SYMMETRY

As stated in the main text, perfect chiral symmetry enforces zero sublattice imbalance for bulk eigenstates in the SSH lattice. In our experiments, the soliton breaks chiral symmetry via the potential it generates, but only locally. In order to quantify the degree of chiral symmetry breaking, we calculate the sublattice imbalance of the eigenstates of the nonlinear Hamiltonian (Eq. 1 in the main text) as a function of power, see Fig. S6. The imbalance for the soliton (red in Fig. 2 of the main text) and topological end state are plotted in red and black respectively, while the blue lines mark the imbalance for bulk eigenstates. For increasing power the imbalance for the bulk states deviates from zero (zero means perfect chiral symmetry), but its deviation is small (below 1%, see inset of Fig. S6, zoomed in on the bulk states) for all soliton powers, indicating that chiral symmetry is nearly preserved for the bulk states.

As stated in the main text, for single-site input we excite a superposition of multiple bulk and possible end states. Therefore, the imbalance for a topologically trivial lattice does not strictly vanish, but decreases as a function of the propagation distance z.

VI. TRANSITION FOR INCREASING J_2

We examine the bifurcation transition as we deviate from a perfectly dimerized lattice ($J_2 = 0$) by increasing the second coupling J_2. Figure S7 shows the transition for multiple values of J_2. For increasing J_2 the bifurcation point of the soliton as well as the transition point of the in-gap state shifts to slightly higher power and the in-gap state's eigenvalue goes to zero more gradually.

VII. COMPARISON WITH A LINEAR DETUNING

For comparison, we calculate the eigenvalues for the case of linearly detuning the potential (i.e. the refractive index) of a single site in the bulk, by replacing Eq. S1 with:

$$E_0 \Psi = H_{\mathrm{lin}} \Psi - \Delta V \Psi \tag{S12}$$

where ΔV corresponds to $g|\Psi_0|^2$ with

$$g|\Psi_0|^2 = \begin{pmatrix} 0 & & & & & & \\ & \ddots & & & & & \\ & & 0 & & & & \\ & & & gP & & & \\ & & & & 0 & & \\ & & & & & \ddots & \\ & & & & & & 0 \end{pmatrix},$$ such that the amount of linear detuning corresponds to the amount of nonlinear detuning if the pump intensity was concentrated on a single site. Interestingly, for linear detuning we observe a gradual decrease of the in-gap state's eigenvalue towards zero (see Fig. S8), while for the nonlinear case this transition is step-like.

VIII. ADDITIONAL MEASUREMENTS

Fig. S9 shows the output intensity when probing the waveguide to the left of the pumped waveguide, site -1 (i.e., its weakly coupled neighbor). Since it cannot host a topological end state, we do not observe an intensity imbalance across A and B sites.

In Fig. S10 we show additional measurements at different positions in the lattice. In all cases, for high input power the intensity in the B-sites to the right of the probe waveguide is noticeably higher than the intensity in the A-sites.

When pumping site B (instead of A) and probing site A (instead of B), we observe that the power threshold for the bifurcation noticeably increases (Fig. S8). Experimentally, we achieve this by keeping our beams

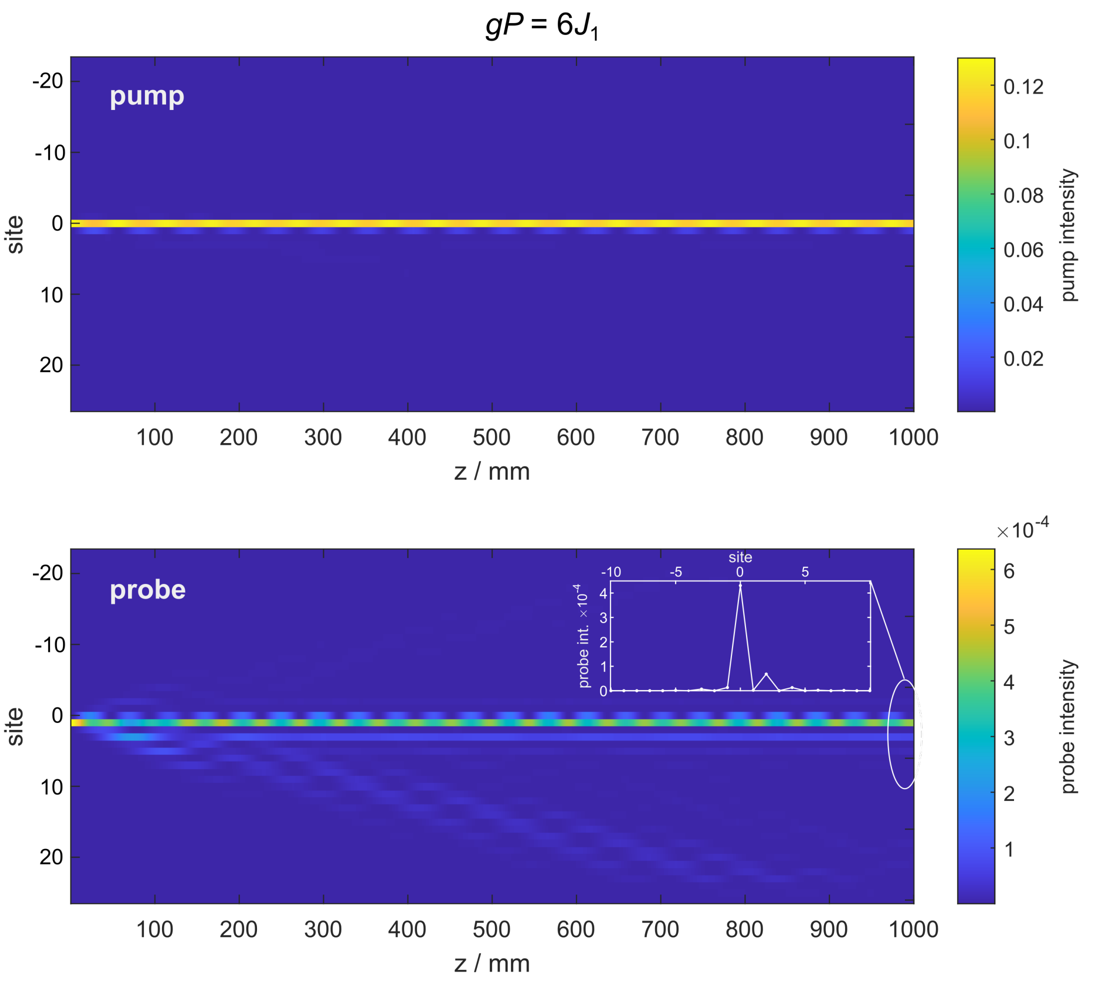

FIG. S5. **Runge Kutta simulations of Eq. (1) and (2) of the main text for longer propagation distances and a power above the bifurcation threshold.** The pump beam is focused into site 0 (upper panel) and the probe beam samples site 1 (lower panel).

identical, but flipping the sample by 180° around the z-axis. This increase of the bifurcation threshold points to a slight on-site potential difference between A and B waveguides due to fabrication. In the Runge-Kutta simulations (Fig. S12), we therefore included an onsite potential of $\Delta = +0.8J_1$ $(-0.8J_1)$ on the A (B) sites. Fig. S13 shows that the onsite potential has negligible effect on the calculated intensity profile of the in-gap state. One should note that additional effects, such as losses in the waveguides and the fact that part of the input light consists of lower power tails of the input pulse, are not captured in simulations.

[1] S. Mukherjee and M. C. Rechtsman, "Observation of Floquet solitons in a topological bandgap," *Science*, vol. 368, no. 6493, pp. 856–859, 2020.

[2] P. Kevrekidis, *The Discrete Nonlinear Schrödinger Equation: Mathematical Analysis, Numerical Computations and Physical Perspectives*, ser. Springer Tracts in Modern Physics. Springer Berlin Heidelberg, 2009.

[3] J. Eilbeck, P. Lomdahl, and A. Scott, "The discrete self-trapping equation," *Physica D: Nonlinear Phenomena*, vol. 16, no. 3, pp. 318–338, 1985.

[4] R. A. Vicencio and M. Johansson, "Discrete gap solitons in waveguide arrays with alternating spacings," *Phys. Rev. A*, vol. 79, p. 065801, 2009.

[5] A. Kanshu, C. E. Rüter, D. Kip, V. Shandarov, P. P. Beličev, I. Ilić, and M. Stepić, "Observation of discrete gap solitons in one-dimensional waveguide arrays with alternating spacings and saturable defocusing nonlinearity," *Opt. Lett.*, vol. 37, no. 7, pp. 1253–1255, 2012.

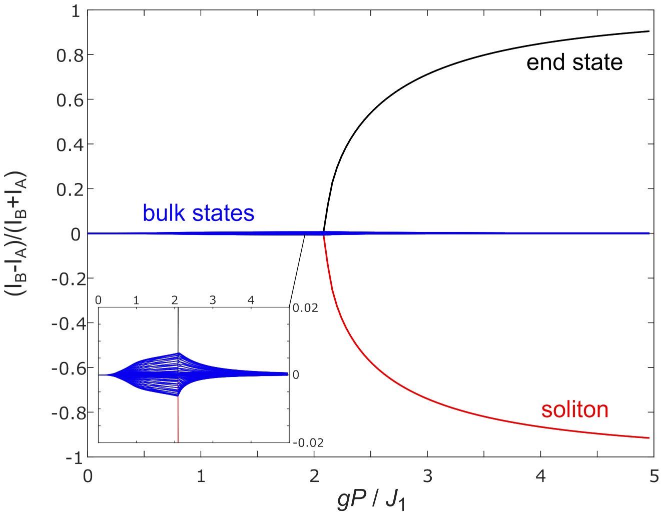

FIG. S6. **Approximate chiral symmetry.** Sublattice imbalance of the eigenstates of the nonlinear Hamiltonian (Eq. 1 in the main text) as a function of power. The imbalance for the soliton is plotted in red, the topological end state in black, and bulk states in blue. The imbalance for the bulk states is not exactly zero, as in the linear case, but very small (see also inset for same data, zoomed in on the bulk states), indicating that chiral symmetry is nearly preserved for the bulk states.

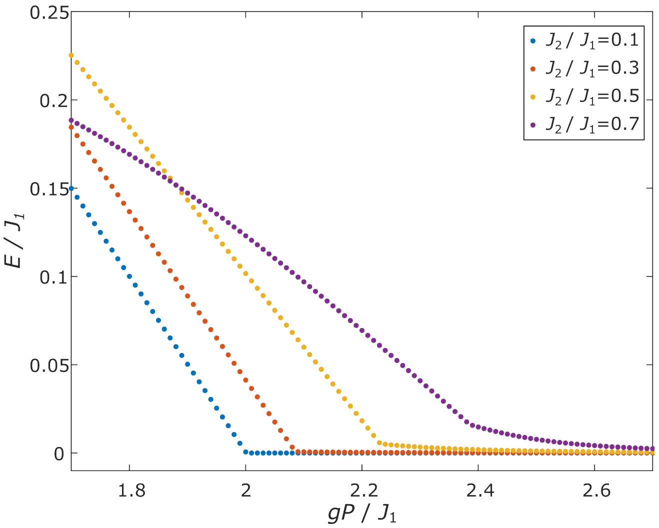

FIG. S7. **Bifurcation for increasing J_2,** zoom-in on the in-gap eigenvalues. For increasing J_2 the bifurcation point of the soliton as well as the transition point of the in-gap state shifts to higher power and happens more gradually.

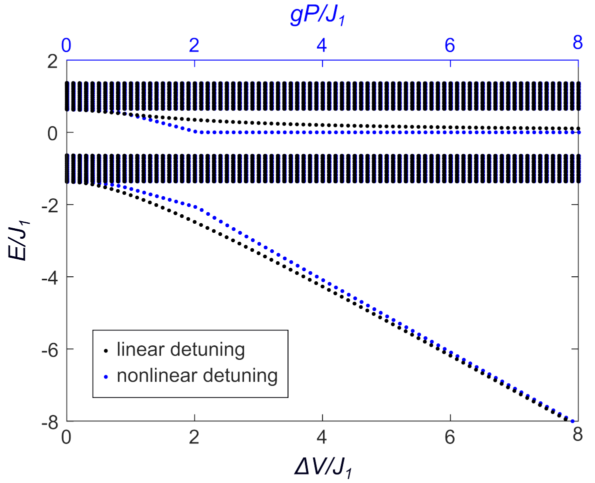

FIG. S8. **Comparison of linear (black) and nonlinear (blue) detuning of sites** and their influence on the midgap eigenvalues, for $J_2/J_1 = 0.37$.

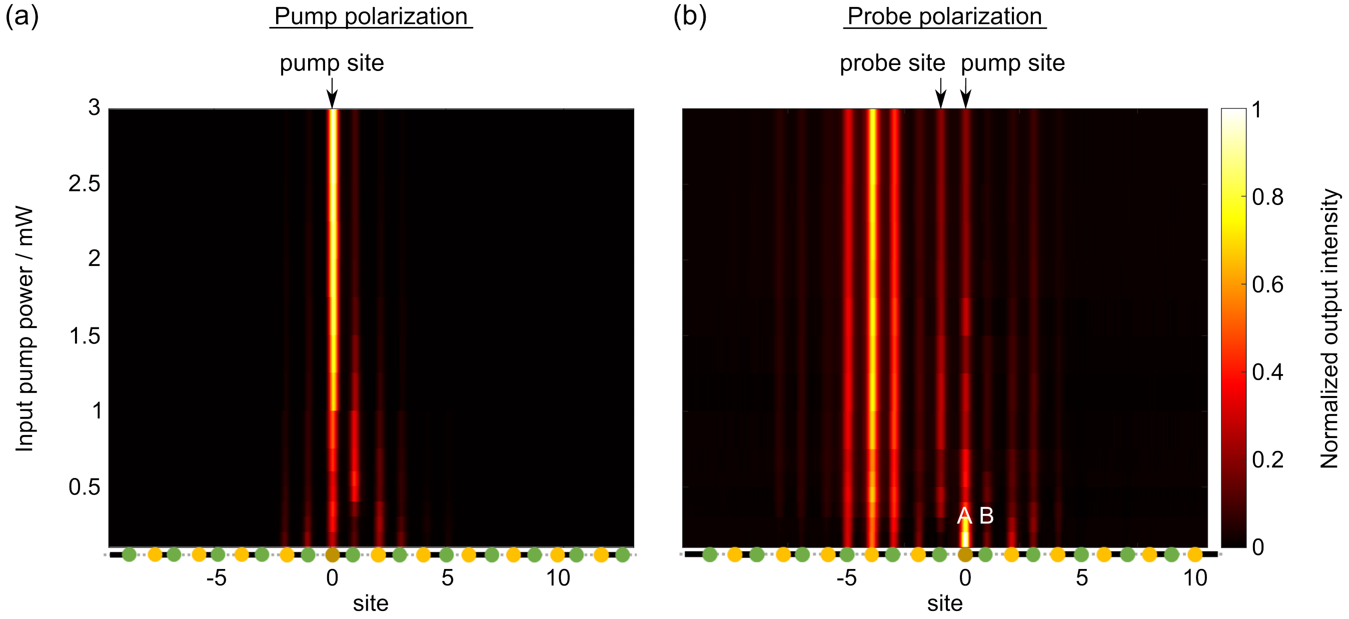

FIG. S9. **Probing the trivial ending** by probing waveguide -1 to the left of the induced soliton. (a) Pump polarization, (b) probe polarization.

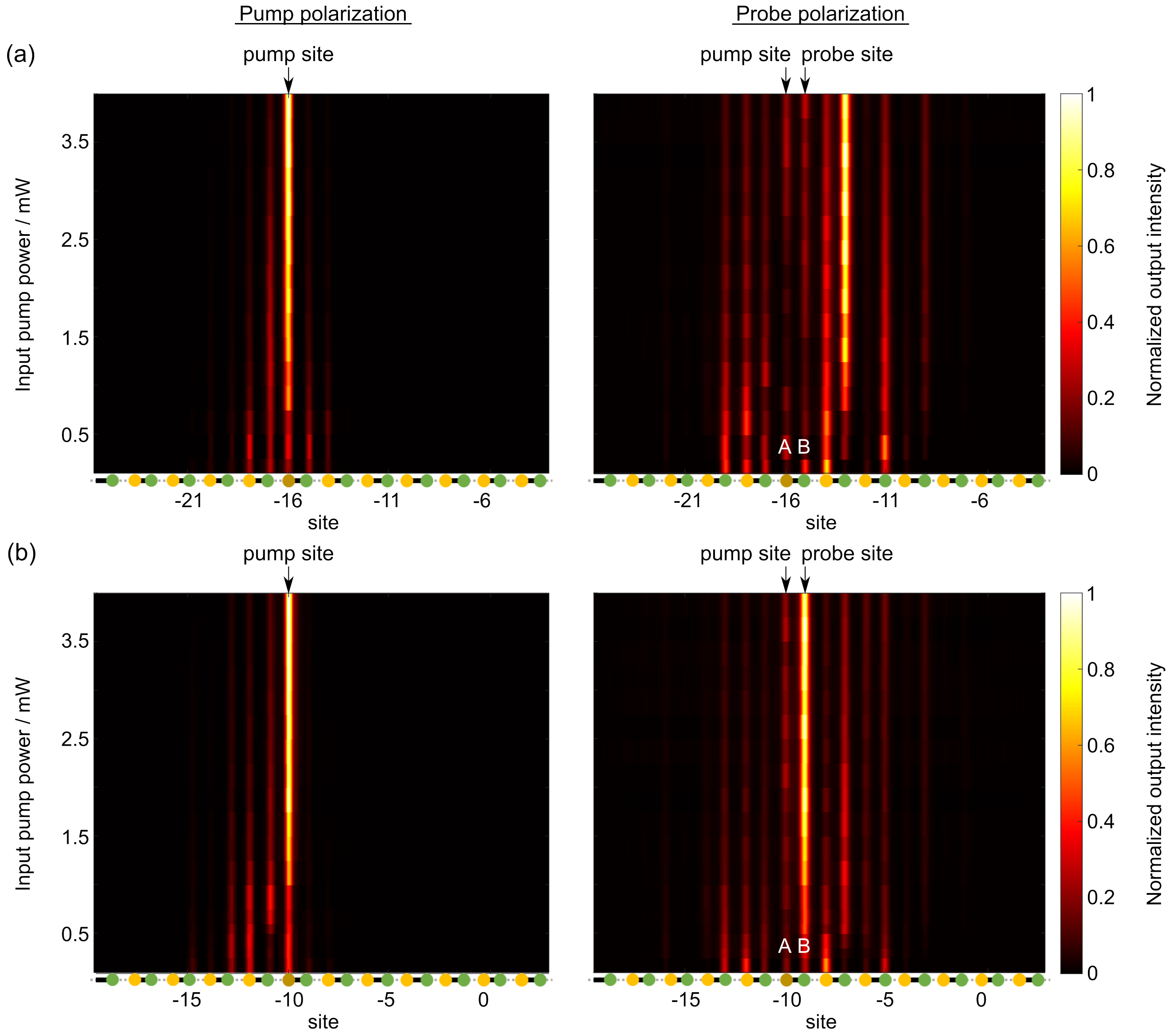

FIG. S10. **Additional measurements at different positions in the lattice.** Left: Pump polarization, right: probe polarization. (a) and (b) are two different measurements at different positions in the lattice, conducted the same way as for Fig. 3 of the main text.

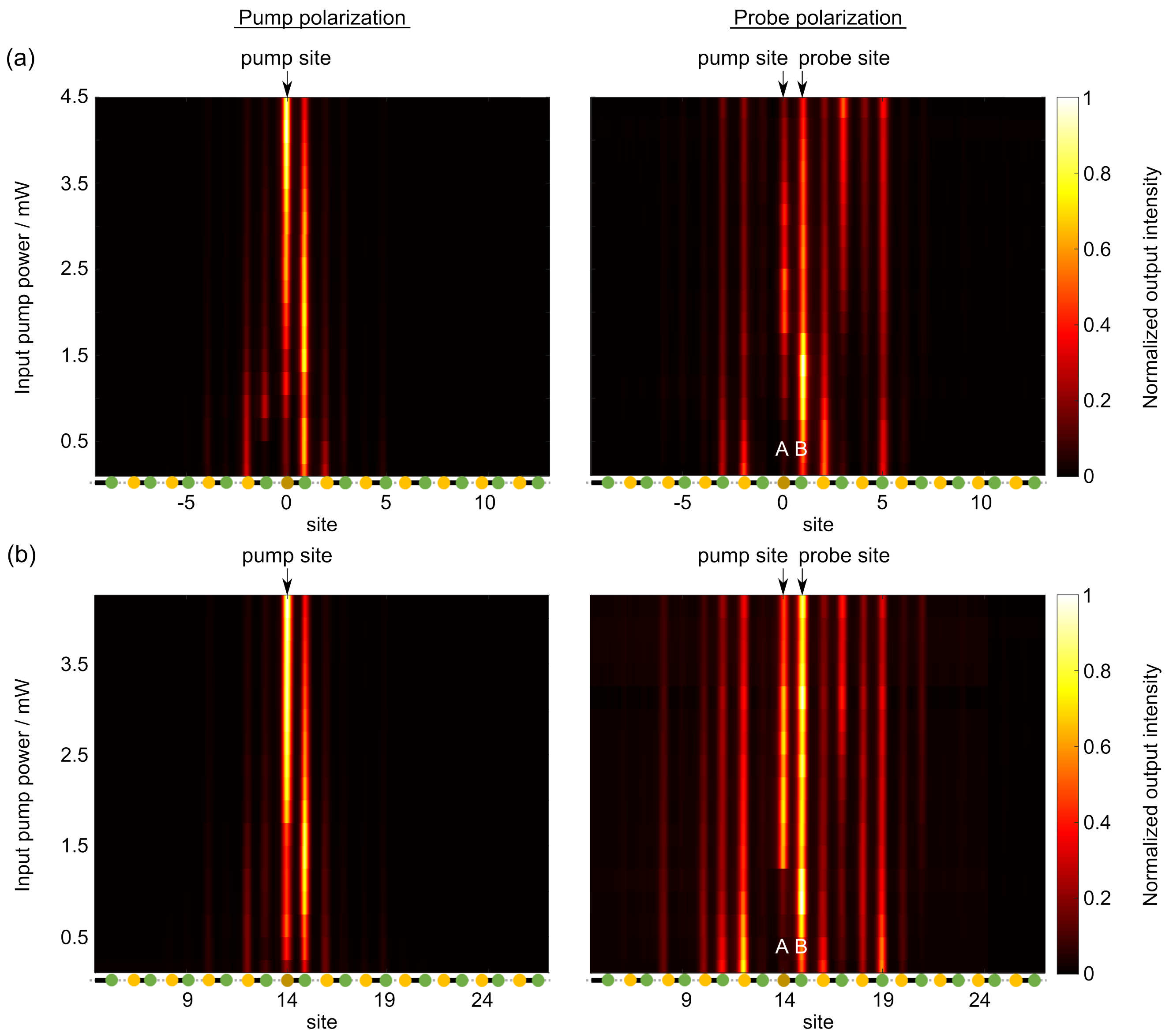

FIG. S11. **Measurements when sample is flipped by** 180° **around the z-axis**. Due to fabrication, one of the sublattices has a slightly higher refractive index than the other, which results in a higher power for the bifurcation threshold when the site with lower refractive index is pumped, as done here (compare Fig. S10). Left: Pump polarization, right: probe polarization. (a) and (b) are two different measurements at different positions in the lattice.

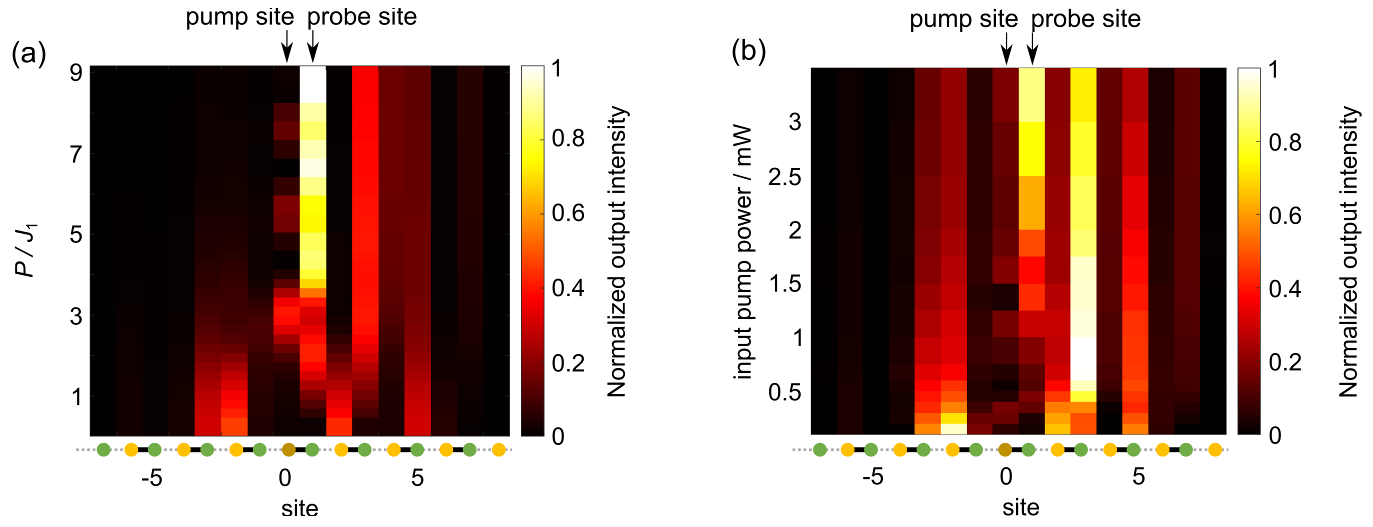

FIG. S12. **Comparison of Runge-Kutta simulations (a) with the experimental data (b).** Shown is the output intensity in the probe polarization. The Runge-Kutta simulations contain an on-site potential of $+0.8J_1$ $(-0.8J_1)$ on the A (B) sites. In (b) only the peak intensity per waveguide site is plotted.

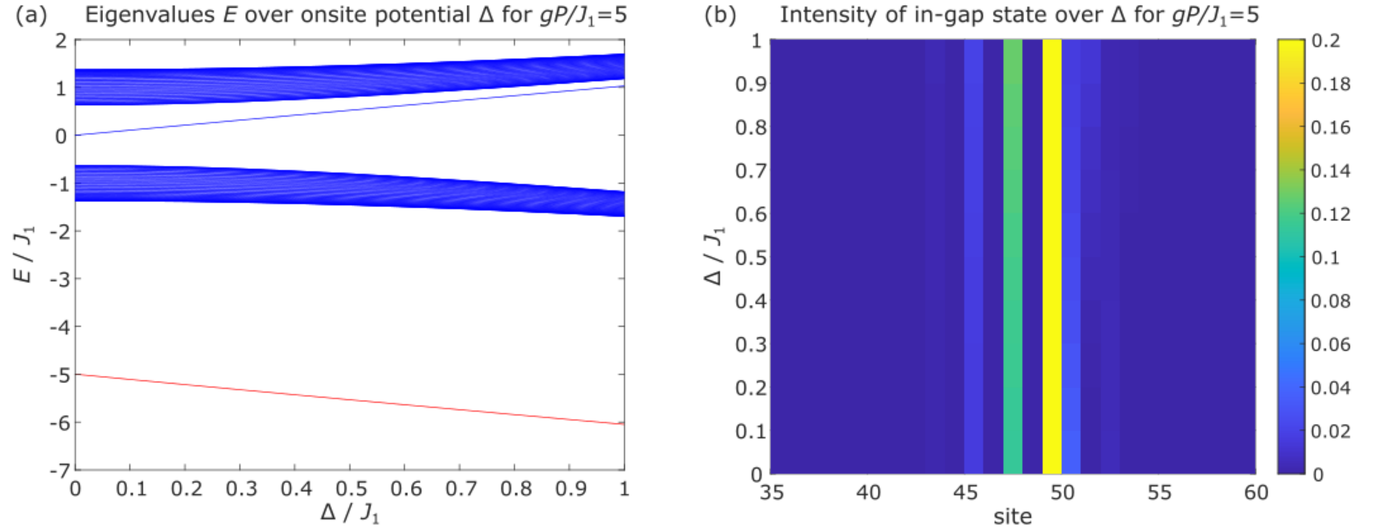

FIG. S13. **Influence of the onsite potential difference:** (a) The eigenvalue of the in-gap state increase from zero (mid-gap) for increasing onsite potential Δ/J_1, while the intensity profile of the in-gap state stays almost the same (b). Both plotted for a power value of $gP/J_1 = 5$, well above the bifurcation point.
